# “You Shall Not Pass” without a Jab: An Institutional Theory Perspective to COVID-19 Vaccine Passport Policies

**DOI:** 10.3390/ijerph192114105

**Published:** 2022-10-28

**Authors:** Emmanuel Ogiemwonyi Arakpogun, Padmali Rodrigo, Femi Olan

**Affiliations:** Newcastle Business School, Northumbria University, Newcastle upon Tyne NE1 8ST, UK

**Keywords:** COVID-19, health crisis, institutional theory, social exclusion, vaccine passport

## Abstract

The recent health crises (e.g., COVID-19, Ebola and Monkeypox) have pointed out huge disparities in vaccine accessibility across the world. Nonetheless, certain governments have instituted vaccine passport policies (VPPs) to manage public health, raising mixed concerns from the public. Focusing on COVID-19 outbreak as an example, this review and commentary article utilises an institutional theory perspective to uncover the factors contributing to the global vaccine divide. We also explore the wider impact of VPPs to determine whether such tools promote freedom or social exclusion. Our insights shed light on a controversial and increasingly divisive policy with an international dimension and institutional implications. For instance, while some argue that VPPs may be relatively better than the blunt instrument of lockdowns, VPPs also implicate access and discrimination concerns. Given the various reasons for global vaccine disparities, a hybrid policy that combines vaccine passports with other public health practices (e.g., rapid lateral flow/affordable polymerase chain reaction (PCR) tests and good hygiene) may be more viable. Furthermore, while VPPs may not be desirable and acceptable domestically, they may be inevitable for international travel.

## 1. Introduction

The COVID-19 outbreak was declared a pandemic on 11 March 2020 by the World Health Organization (WHO) [1]. On the other hand, on 13 May 2022, the WHO reported several cases of monkeypox across Africa, America and Europe [2]. While several vaccines have been developed in response to COVID-19, vaccines have also been approved for the prevention of Monkeypox outbreaks [2,3]. Typically, the development of vaccines takes a minimum of 10 years, although the mumps and Ebola vaccines took four and five years, respectively [4,5]. In the case of COVID-19, multiple vaccines were granted emergency approval in at least one country, including Pfizer-BioNTech, Oxford-AstraZeneca, Sputnik V, Johnson & Johnson, Moderna and CoronaVac by July 2021. As of that same date, over 3.1 billion vaccine doses were administered globally, which is more than 17 times the global infection rate of over 183 million [6]. As of 19 August 2022, over 12 billion COVID-19 vaccine doses have been administered globally. However, data from relevant sources (e.g., *Financial Times*, Hopkins University & Medicine and Our World in Data) indicate that the majority of countries that have administered over 60% of global jabs (including multiple doses and boosters) are located in advanced countries such as those in the European Union, the UK and the USA—a clear indication of a large vaccination divide.

Governments sometimes mandate certain measures and behaviours such as lockdowns to reduce harm [7,8,9,10]. In the midst of the global vaccine divide, *vaccine passport policies* (VPPs) were proposed as a potential measure to encourage people to take vaccines [11,12,13]. In this review, we focus on vaccine passports offered for COVID-19. A vaccine passport, in this case, is a formal proof that someone has been vaccinated against an infectious outbreak. It is argued that VPPs should be integrated into public health management, so that vaccine passports can be used to access domestic and international socio-economic activities curtailed by an outbreak such as COVID-19. For example, Israel was one of the earlier countries to implement a domestic VPP, which involves loading a COVID-19 ‘green pass’ onto smartphones and issuing physical cards 14 days after the second dose to enable access to public places such as cultural events, gyms and restaurants [14]. Furthermore, Israel also explored an international VPP with Cyprus and Greece, to allow vaccine passport holders from Israel to holiday without self-isolation upon arrival [15]. The government of Qatar has suggested that only fully vaccinated football fans will be allowed at the FIFA 2022 World Cup in November 2022 [16].

Similar policies were implemented/planned across Europe. For example, a vaccine passport was required for football fans to watch Euros 2020 matches at Wembley stadium in the UK. While the UK government initially announced that a vaccine passport would be needed for public places such as pubs, they backtracked on this requirement following public outcry, the revolt of 70 cross-party parliamentarians and an opposing public petition that generated over 200,000 signatures [17]. From the travel industry (e.g., British Airways and SAGA) to dating apps (e.g., Bumble and Tinder), non-state actors across the UK also adopted some form of VPPs [18,19,20]. Elsewhere in Europe, the EU launched ‘the EU Digital Certificate’ to facilitate ‘safe’ and unrestricted travel across its 27 Member States for vaccine passport holders [20,21]. More than 25 countries have considered VPPs [22]. See Table 1 for more examples of state and non-state actors’ actions on VPPs.

Regardless of the country and phase of implementation, VPPs have raised serious concerns. For example, while a former UK vaccine minister once argued that it would be ‘remiss’ for the government to ignore VPPs as a tool for fully reopening the economy, a former Prime Minister (Boris Johnson) admitted that VPPs raise deep and complex ethical issues that need to be explored [23]. For example, to what extent can such a passport be an effective measure for several types of outbreaks that we face on a regular basis? This raises a series of research questions:

1. What are the key factors responsible for the global vaccine divide?
2. Why do some people support VPPs and others oppose them?
3. What are the wider implications for VPPs?
4. How can governments increase public support for COVID-19 vaccination and VPPs?

To answer these questions, we adopted an institutional theory perspective to examine academic literature and secondary data drawn from online newspapers and publicly available survey data from Ipsos MORI about global perceptions of COVID-19 VPPs. We found that although VPPs play a role in helping government and public health authorities better manage COVID-19, VPPs could also lead to discrimination and social exclusion.

## 2. Materials and Methods

To better understand the VPPs debate and answer the four research questions raised in Section 1, this review draws on publicly available data from the Ipsos MORI survey commissioned by the World Economic Forum of 21,000 participants across 28 countries (e.g., Argentina, China, France, South Africa, Saudi Arabia and the US) [24]. Ipsos MORI released this survey at the end of April 2021—a period that coincided with an increasing number of countries proposing and trailing VPPs. Among other issues, the survey focused on participants’ perceptions of COVID-19 VPPs in relation to, for example, opening up social activities and going back to workplaces. To further triangulate the Ipsos MORI survey and to keep track of COVID-19 policy changes across various countries, we tracked, documented and drew more evidence from academic literature and secondary sources such as online media and newspapers.

For example, Euro News, Financial Times, John Hopkins University COVID-19 resource center and Our World in Data was constantly consulted for country-by-country performance for vaccination. Airfinity, the Launch and Scale Speedometer, NPR.org and Oxfam International were used to ascertain COVID-19 vaccine stock per country. We also tracked BBC News, CNN, Chatham House, *The Economist*, *Financial Times*, The Conversation and various government COVID-19 daily press conferences to keep on top of emerging issues related to, for example, VPPs, travel restrictions, lockdown policies, mask mandates, etc. These secondary data sources were mainly tracked from 1 April to 1 October 2021.

## 3. Results

### 3.1. Tracking COVID-19 Vaccination Rollout and its Hindrances

Although over 12 billion COVID-19 vaccine doses were administered globally as of 19 August 2022, over 60% of these doses have been administered in advanced countries (e.g., European Union countries, Israel, the UK and the USA)—implying that over 100 countries are lagging behind. Such global vaccine divide is concerning, particularly given the growing consensus that vaccines are the most viable strategy for effectively managing COVID-19 pandemic [25]. This is consistent with the argument by Ursula von der Leyen, the President of the European Commission, that ‘… a global pandemic requires a world effort to end it—none of us will be safe until everyone is safe’ [26].

While countries such as China, India and the US are among the leading countries on the total number of doses administered, the data for most developing countries are not encouraging. For example, in Nigeria—the most populous African country with over 200 million people—only 13.7% of the population is fully vaccinated at the time of writing, indicative of most developing countries across Africa and Asia. Therefore, global vaccination data suggest wide disparities among regions and countries.

**Figure 1:**
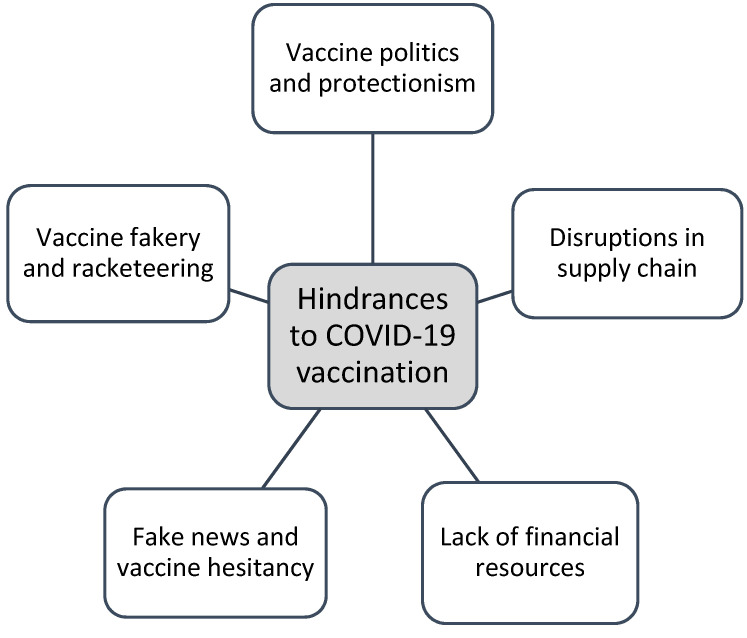
Hindrances to COVID-19 vaccination.

Thus, an investigation into hindrances to the COVID-19 vaccine rollout is urgently needed. Figure 1 provides a broad categorisation of the multiple issues fuelling inequitable COVID-19 vaccination.

#### 3.1.1. Vaccine Politics and Protectionism

First, vaccine politics and protectionism are fuelling the global vaccine divide. As global demand continues to outpace supply, vaccine shortages are creating political tensions worldwide. For example, Italy blocked the export of 250,000 Oxford-AstraZeneca doses from being shipped to Australia [27]. The EU has also threatened to block the export of vaccines to the UK twice in response to its vaccination delay, despite having over seven million doses of Oxford-AstraZeneca stored across the EU as vaccine hesitancy increases. Former US President Donald Trump signed an executive order in December 2020 to ensure that vaccines made in the US were first administered for domestic use before export [28]. The Indian government has curtailed the export of Oxford-AstraZeneca vaccines produced at the Serum Institute to ensure local administration [29], a policy impacting vaccination efforts in over 70 countries, including developing countries.

#### 3.1.2. Disruptions in the COVID-19 Vaccine Supply Chain

Given that vaccine production stretches across multiple countries, the emergence of vaccine politics and protectionism has disrupted the global COVID-19 vaccine supply chain. For example, although the Serum Institute of India was contracted to produce 240 million doses of the Oxford-AstraZeneca vaccine for developing countries, the majority of the EU’s 400 million doses were produced in plants across the US and in Belgium [28,29]. Furthermore, the entities contracted to produce the vaccines are usually not located in the same country as those responsible for the vials (filling, bottling and finalizing for export). According to Danny Hendrikse (Pfizer’s VP for global supply), 280 pieces of raw materials from over 80 suppliers across 19 countries are required to produce a single dose of Pfizer-BioNTech vaccine [30]. Similarly, the US Moderna vaccine for Europe is produced in Switzerland, while the vials are produced in Spain [29]. As vaccine politics and protectionism increase, certain countries may retaliate for not receiving what was promised by withholding vaccine components. This will throw the global vaccine supply chain into chaos, setting back many countries’ vaccination programmes.

#### 3.1.3. Lack of Financial Resources

A third hindrance is the lack of financial resources to secure adequate vaccine supplies, especially for developing countries. Vaccine development typically involves huge financial investments in R&D [31]. Moreover, the scientific and manufacturing capabilities of vaccine production are often concentrated in advanced countries, where the government is the major financier of ‘proof of concept’. For example, Moderna received approximately USD 4 billion to support its proof of concept, while Pfizer-BioNTech received USD 1.95 billion from an advanced purchase agreement via Operation Warp Speed under the Trump administration [31]. Overall, the US invested over USD 10 billion into the production and procurement of COVID-19 vaccines. While it is unclear how much the Chinese and Russian governments spent on their vaccine candidates, the German government invested about USD 445 million into Pfizer-BioNTech, while the UK government gave over USD 65 million to Oxford University for the Oxford-AstraZeneca vaccine [31].

Consequently, vaccine demand, supply and pricing are determined by a small group of actors or nations. For example, Oxfam [32] found that from a projected 5.94 billion doses of vaccines produced from 2020 to 2021, over 2.7 billion doses were bought by developed countries before production, which is more than 50% of the global stock for the same period.

**Figure 2:**
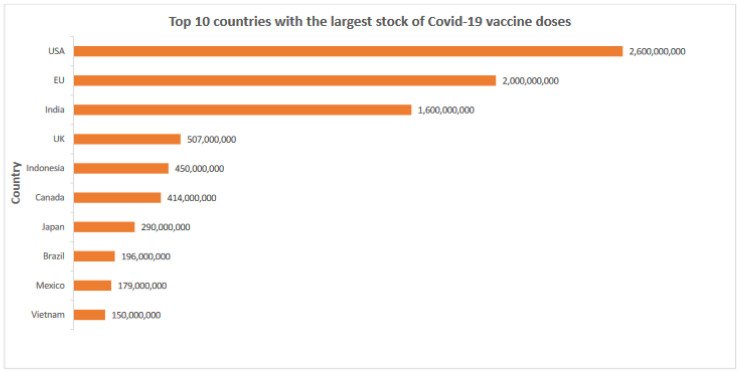
Top ten countries with the largest number of COVID-19 vaccine doses ordered. Source: Compiled by the authors from a variety of sources including Airfinity, Launch and Scale Speedometer, NPR.org, and Oxfam International.

Figure 2 underlines the argument that a market-based model for vaccines may not benefit global public health [31], as wealthier countries will acquire the majority of the output, leaving developing countries behind. The Vaccine Alliance (Gavi) and the Coalition for Epidemic Preparedness Innovations (CEPI) instituted COVID-19 Vaccines Global Access (COVAX) to mitigate the impact of vaccine affordability and shortages for developing countries. COVAX has raised over USD 6 billion to distribute 2 billion COVID-19 vaccine doses to 92 developing countries in 2021 [31,33]. However, the emergence of vaccine politics and protectionism threatened the objective of COVAX and vaccination programs in developing countries.

#### 3.1.4. Fake News and Vaccine Hesitancy

Fake news—the deliberate (disinformation) or unintended (misinformation) proliferation of falsehoods about a given topic—has eroded public trust in the safety of COVID-19 vaccines. Rodrigo et al. [34] argue that COVID-19 and the ubiquity of technology have given rise to an infodemic of falsehoods, where communities and individuals knowingly and unknowingly share inaccurate COVID-19 information. For example, WHO [35] curated a series of ‘mythbusters’ to counter falsehoods, such as 5G networks being responsible for COVID-19, drinking bleach cures or prevents COVID-19, and the ability to hold one’s breath for more than 10 s is a sign one is not infected. Thompson [36] also documented the negative impact of the spread of vaccine falsehoods among people in their 20s and 30s in Japan, which has shifted toward vaccine hostility [36], thereby increasing vaccine hesitancy. France has become one of Europe’s most vaccine-hesitant countries, partly because of the proliferation of fake news by anti-vaxxers [37]. Only 59% of the population in France was willing to be vaccinated, which is lower than in China and the UK, where 80 and 70% of the population, respectively, were willing to be vaccinated [38,39,40].

#### 3.1.5. Vaccine Fakery and Racketeering

The fifth hindrance is vaccine fakery and racketeering. While the former refers to access to fake vaccines, the latter refers to illegal access to authentic vaccines. Public authorities across China, Nigeria and South Africa have confiscated over 8000 fake COVID-19 vaccine doses [40,41,42]. The possibility of counterfeit COVID-19 vaccines raises public anxiety and public health concerns. Similar to the discovery of vaccine fakery in both developing and developed countries (e.g., *The Guardian* [43] for an incident in the UK), vaccine racketeering has emerged in both contexts. For example, Nigerian authorities reported a racketeering scheme where corrupt individuals at inoculation centres provided vaccines to individuals outside the government’s top priority list of recipients [44]. In Italy, of the 140,000 doses administered by the first week of April 2021, thousands were administered to non-healthcare individuals who did not fall under the targeted priority medical worker [45]. Such corrupt practices can further erode public trust, a critical element in a vaccination campaign [46].

### 3.2. The Vaccine Passport: A Tool to Freedom or Social Exclusion?

While COVID-19 VPPs are increasingly divisive, evidence from our secondary sources indicates that vaccine passports are not new. For example, while Chadwick et al. [47] explored vaccine passports as a tool for increasing vaccination for people living with HIV (PLWH), Grobusch et al. [48] revisited the introduction of yellow fever vaccine certificates over a decade ago. Currently, travellers to countries with yellow fever are required to show proof of vaccination, which is typically recorded on a physical (‘yellow card’) certificate [12,49]. However, unlike COVID-19, the yellow fever vaccine is readily available, and yellow fever is endemic in tropical countries across Africa and Latin America [49,50]. Furthermore, a single dose of the yellow fever vaccine is sufficient for lifetime protection [50].

Given the global impact of COVID-19 and its associated intractable issues, it is not surprising that VPP proposals have divided public opinion. We draw from the Ipsos MORI survey along with academic literature to better explain the VPP debate. Table 2 provides the key highlights of this data relevant to our review.

Proponents view vaccine passports as a tool for managing COVID-19 risk as countries emerge from lockdowns. With vaccine passports, it is easier to identify those who are less likely to spread infection and to suffer from adverse effects of COVID-19. For example, Kirsty Innes, the head of Digital Government at the Tony Blair Institute, argued that vaccine passports can be required for care home visits to mitigate the impact of COVID-19 on residents, who are particularly vulnerable to COVID-19 [51].

Furthermore, vaccine passports are viewed as a safer way to reopen society and to reinstate civil liberties. They may be a more suitable tool for mitigating COVID-19 contagion than blunt instruments such as lockdowns, which caused over USD 12 trillion in cumulative losses to the global economy from 2020 to 2021, the loss of over six months of education for some children [31,52] and a drastic reduction in the bottom line of businesses across the globe [53]. Proponents argue that if VPPs can end lockdowns, such policies should be encouraged and embraced to accelerate a return to normalcy with less risk to public health. Furthermore, VPPs may offer a ‘smart’ alternative to lockdowns with a view to pre-empting the impact of different waves [54] and new variants.

Conversely, critics such as Silkie Carlo, Director of Big Brother Watch, UK, argue that VPPs could encourage state surveillance and could endanger data privacy [51] given that digital vaccine passports may contain personal health records and other identifiers that can be mapped, stored and tracked [55,56]. Moreover, digital passports raise concerns about the government’s ability to ensure the safety and security of public health data. For example, Singapore introduced digital contact tracing to track COVID-19 infections. While it was promised that the data gathered would only be used to curtail COVID-19, the Singapore government later admitted that police can access user data from the TraceTogether app ‘for criminal investigations’ [57].

VPPs can lead to discrimination and segregation as the unvaccinated in society will be excluded from socio-economic activities, including those unable to receive the vaccine for various reasons. For example, the low vaccination rate among ethnic minorities in the US is linked to the lower priority given to younger age groups, to which most ethnic minorities belong [58]. Moreover, the vaccination rate among White people is double the rate of African and Hispanic Americans [59]. Other critics argue that under current UK law, VPPs will result in indirect discrimination against ethnic minorities who are currently less inclined toward vaccination in the UK [60]. As a result, VPPs can create a ‘two-tiered’ society where, for example, the over 50s are free to lead ‘normal’ lives while other age groups are disproportionately impacted as they wait for their shots.

Furthermore, VPPs can be an indirect way of introducing mandatory vaccination because people may be indirectly ‘coerced’ into becoming vaccinated to avoid discrimination and missed opportunities. For example, some people in Israel admitted that they received the vaccine to avoid being denied access to public places, social activities and international travel [58]. Effectively, VPPs exert pressure on people to receive the vaccine; this goes against international conventions such as the Council of Europe (p. 3, [61]), which stipulates that governments should “ensure that citizens are informed that the vaccination is NOT mandatory and that no one is politically, socially, or otherwise pressured to get themselves vaccinated if they do not wish to do so themselves; ensure that no one is discriminated against for not having been vaccinated, due to possible health risks or not wanting to be vaccinated”.

Another area of concern is employment. Some sectors considered VPPs for employees. For example, care homes and plumbing firms in the UK suggested that new employees will need to provide evidence of vaccination, while Italy became the first European country to mandate vaccines for healthcare workers [58,62]. Given that over 3000 people died of COVID-19 after contracting the disease in hospitals across the UK [63], it may be logical to mandate vaccination for healthcare workers to protect both staff and patients. However, a wider vaccination mandate as a condition for employment could cause difficulties for certain people who have been disproportionately impacted by COVID-19, including ethnic minorities, individuals from low-income households and other vulnerable groups [64,65]. Therefore, mandating vaccination for employment purposes could lead to unintended consequences by further entrenching existing structural inequalities and untold harm to vulnerable groups.

Critics further argue that COVID-19 vaccines are effective in breaking the link between serious illness and death, but not for transmission, as vaccinated individuals can still contract and spread COVID-19. Indeed, the WHO is against VPPs due to the limited global supply of vaccines and the lack of evidence on the vaccine’s ability to stop COVID-19 transmission [58]. Moreover, as leading vaccination countries such as Israel, the US and the UK raced ahead, large parts of the developing world struggled to receive an adequate vaccine supply. Thus, while vaccine passports may allow people from advanced countries to engage in international travel, vaccinated travellers could be impacted by local outbreaks in visited countries or could contract new variants.

The emergence of new variants and vaccine politics suggest that some countries would inevitably have vaccine preferences. For example, the emergence of the Alpha (B.1.1.7 first detected in the UK), Beta (B.1.351 in South Africa), Gamma (P.1 in Brazil) and Delta (B.1.617.2 in India) variants have raised concerns about the efficacy of existing vaccines [66]. For instance, although South Africa participated in the trial phases of the Oxford-AstraZeneca vaccine, the country now favours the use of Pfizer-BioNtech and Johnson & Johnson vaccines, which are believed to offer better protection against the Beta variant [67]. Furthermore, because different governments are major R&D contributors to existing vaccines, some governments may prefer their vaccines to promote either public trust or ‘soft power’ worldwide. For example, China has insisted that it would be easier for foreigners who have received the Chinese vaccine to obtain their visa [68]. The EU reportedly will not recognise Russia’s Sputnik V, China’s CoronaVac or Oxford-AstraZeneca made in India’s Serum Institute.

This raise concerns over the acceptability of vaccine passports and the implications for travellers who have received vaccines that are not acceptable in certain countries. Perhaps this is one area of difficulty where the international collaboration previously indicated in Section 4 could help to mitigate. Moreover, vaccine politics underscored here indicates that it is not sufficient for the global scientific community to produce vaccines for a given pandemic; international collaboration is further needed for a worldwide consensus for vaccine acceptance [69] with a view to overcoming the unacceptability of certain vaccine passports. This position is further supported by Perez and Abadi (p. 574 [70]), who stated that “Defeating this pandemic is impossible without united and coordinated international attempts shaped by all countries of the world”.

## 4. Discussion of the Wider Complexities Associated with Vaccine Passports and Implications for COVID-19: An Institutional Theory Perspective

To discuss the complexities associated with mandatory VPPs along with the theoretical and practical implications of our results, we adopt an institutional theory perspective. Institutional theory suggests that a variety of actors within a market construct and shape the meaning of consumption practices and influence the way a market develops and evolves [71]. Typically, consumers (the public in this case) are viewed as passive meaning takers (with no ability or opportunity to resist) or proactive meaning makers or givers [72]. Meaning makers or givers are called institutional entrepreneurs because they are expected to shape the institutions that determine consumption practices in the market [71]. However, institutional entrepreneurship can only occur in markets where consumers can resist the consumption offerings provided by powerful actors. Thus, institutional theory provides insights into how macroprocesses shape the behaviours of organisations and the environment. To provide an informed contribution to the current polarising debate around VPPs, institutional theory can be useful in gaining insights into public behaviour and reaction.

The ongoing debate around COVID-19 VPPs is based on market conditions where consumers (i.e., the public) are ‘forced’ to take the vaccine to meet the powerful actors’ expectations. Powerful actors, in this case, include state (e.g., governments and public health authorities) and non-state (e.g., lobbyist groups such as the travel and sports industry) actors. Accordingly, consumers are forced to become *passive meaning takers* rather than *meaning makers or givers* as ‘they are left with no choice’ but to receive the vaccine. Even if consumers are willing to receive the vaccine, they are not allowed to choose what vaccine they will receive (e.g., Oxford-AstraZeneca vs. Pfizer-BioNTech) or when and where they will receive it. This is supported by the highlights listed in Table 1.

However, several issues need to be considered carefully before implementing VPPs: Will the public resist VPPs? What possible complexities does one need to overcome when introducing vaccine passports such as public hesitance, and what consequences can such resistance create in the short and long term? How can institutions address this resistance? From a consumer perspective, it is important to understand the potential barriers to public acceptance and rejection of vaccination in a manner that avoids social exclusion. Understanding how and why consumers resist and how institutional actors address these concerns will play a vital role in successful VPP implementation. Theories on consumer resistance and institutional theory can shed some light on this issue. Hirschman [73] argues that in a general consumption situation, if consumers are dissatisfied with a company offering, they are often able to react to by avoiding or rejecting the offer. Specifically, Hirschman [73] states that when consumers are dissatisfied, they can exit, voice or express their disloyalty: exit means leaving the firm and voice means expressing themselves by protesting the firm. According to consumer resistance and institutional theory, despite dissatisfaction, consumers may still tolerate and accept discontent if they are allowed to voice their concerns [71]. However, when consumers are unhappy with a firm’s offer with no avenue to express their dissatisfaction [74], they can adapt a range of reactions spanning from avoidance to rebellion.

In the context of COVID-19, consumers’ freewill is limited. For instance, clinically vulnerable patients may be left with no choice but to receive the vaccine even if they prefer not to because of potential side effects. Such scenarios violate consumers’ freedom to act or resist because the potential consequences of resistance are social exclusion or even their life. Chaney and Slimane [71] identify four types of consumer segments that can resist market offerings: passive consumers, resigned consumers, entryists and rebels, as shown in Table 3. This classification can be adapted to explain expected types of resistance against VPP implementation.

For instance, individuals (*intentional*) without any barriers to consumption but who do not want to consume the vaccine can be classified as rebels and passive consumers. These could be members of the public who are misinformed or consider vaccines to cause long-term side effects. VPPs will exert a force on such consumers because, regardless of their resistance to vaccination, they will be left with no choice but to receive the vaccine to avoid social exclusion. Consistent with the theories of resistance, two categories of consumers can emerge from this situation: (i) those who do not want to receive the vaccine and have a certain propensity to oppose vaccination (rebels) and (ii) those who do not want to receive the vaccine but lack the propensity to oppose vaccination (passive consumers).

The second group of consumers (*non-intentional*) will be those willing to receive the vaccine but who are excluded due to certain barriers. For instance, Section 2 indicates that a few countries dominate the global stockpile of existing COVID-19 vaccines, creating disparities between regions and countries. Young people also face significant social exclusion, as the majority have not been offered the vaccine and are not classed under the priority list. In any case, this second group can be further subdivided into two groups [71]: (i) those who want to receive the vaccine but are excluded due to barriers and then resign themselves to the situation, and (ii) those who want to receive the vaccine but are excluded due to barriers and then try to change the situation. In the first subgroup, the public may react to institutions by either adopting a passive approach or resigning. Such responses may not garner opposition to VPPs or alter the status quo. However, in the second subgroup, due to the presence of resistant actors, the public may act as reflective actors who oppose VPPs implementation.

Rebels refer to those who can receive the vaccine but do not want to because they oppose the market for political, religious, economic, or other reasons [71]. Such reflexivity of rebels is influenced by their oppressive response to established institutions. Conversely, entryists refer to individuals who are unable to consume but wish to do so [71]. In the COVID-19 context, this group could be young people excluded by government policies or individuals with certain medical conditions. This group will perceive that their exclusion is unfair. Rebels and entryists can differ in terms of the nature of change they wish to bring about in their respective environments. However, due to the oppression they feel, rebels and entryists can act in two ways to bring necessary changes. First, they can contest the legitimacy of established practices and deinstitutionalise them, which will cause the acceptance of institutional offerings to collapse. Second, they can create an alternative space to counter the narratives of established institutions.

The existence of such different clusters of consumers with different attitudes towards VPPs and issues associated with an individual’s ability to take a vaccine further raise concerns associated with ethics and equality [61]. Giubilini and Kennedy (p. 1) [75]) argue that despite the ability of COVID-19 vaccines to reduce hospitalisation, deaths and result in a lesser burden on the health system, such promises do not resolve “the question of whether they [VPPs] are ethically justifiable”. Moreover, the existence of different clusters of the public with varied intentions and hesitancy may hinder the ability of relevant policymakers to implement an all-inclusive VPP approach in a manner that respects individuality and their rights to free travel. For instance, the proposed mandatory VPPs could become problematic by violating the right to travel freely for those who are unvaccinated due to medical, religious, or other reasons. Therefore, policymakers need to pay careful attention to the rights of those who cannot be vaccinated. Furthermore, attention should be given to issues associated with discrimination as mandating VPPs could lead to increased freedom for those who are vaccinated compared to the unvaccinated and/or those unable to have the vaccine due to underlying health conditions. As such, rebels and entryists may feel discriminated against due to religious, medical and other reasons.

Additionally, the implementation of VPPs indirectly allows for easy identification of medical conditions to third parties that an individual may be unwilling to share under normal circumstances. For example, the need to shield vulnerable people led to the disclosure of their ‘vulnerability status’ to a wider group that would normally not have access to such medical information. Moreover, those who were needed to be shielded had to share their vulnerable status to receive priority access with supermarkets, energy suppliers, property management companies, etc. This can lead to stigmatization and can impact the privacy, dignity and psychological well-being of those affected.

The equitability of VPP is another issue that needs to be addressed. As mentioned in Section 3, the supply of vaccines is disproportionate. Such existing inequalities in vaccine supply can threaten the equitability of VPPs [76]. As illustrated in Figure 2, countries such as the USA and the UK hold the largest stock of vaccines compared to other countries. Access to vaccines is therefore higher among the countries with larger economic, social and political power leading to an inequality in the supply and distribution of vaccines. Hence, policymakers need to take proactive steps to improve access to vaccines to ensure equality and to mitigate discrimination against those that lack access to adequate vaccine supply [76].

The implementation of VPPs can further be problematic in regions where several humanitarian and economic crises are prevailing (recent examples include Iran and Ukraine). Hesitancy toward VPP can also be higher among individuals that belong to different communities. For instance, some ethnic minority groups have been found to show a high degree of resistance to vaccination [77,78,79]. Moreover, VPP resistance is also higher among communities with lower income and education [80]. This group of people is more prone to believe conspiracy theories [81]. Hence, they tend to distrust governments and health authorities. Distrust, in this case, acts as a barrier to the successful implementation of VPPs.

Given the complexities associated with the implementation of VPPs along with the different prevailing country-level limitations (e.g., limited supply, vaccine hesitancy arising from pre-existing health conditions, religious beliefs, etc.), policymakers need to consider other countermeasures at both the national and international levels to complement VPPs. For example, some scholars, e.g., [82,83] suggest the need for regional cooperation to foster ‘mutual sharing’ in a manner that allows respective regions to leverage their comparative advantage and geopolitical diplomacy to support one another. From an international standpoint, developed countries with more persuasive power and clout could coordinate an international effort with, for example, the WHO to ensure more equal access to vaccines and other relevant resources needed to combat the pandemic. In collaboration with the countries mentioned in Table 1, the WHO also convened a ‘Smart Vaccination Certificate Working Group’ aimed at establishing technical guidance with key specifications and standards, taking into consideration local, national and international challenges [79,83,84,85]. The technical guidance was developed on standards and an adaptable framework that produces a digital vaccination certificate to support the implementation of VPPs across the globe. The Smart Vaccination Certificate Working Group’s agenda was to develop an environment that supports continuity of protection for every community and implementation across borders. Furthermore, the Working Group focuses on a learning community that supports the worldwide adoption of specifications and standards for VPPs, thereby proving a credible alternative to mandatory VPPs. The collaborative efforts suggested could further act as a countermeasure to boost vaccine supply for those countries that are lagging behind. Considering the potential of VPPs to impact peoples’ rights and privileges, policymakers may consider a ‘graduated’ approach [86] to VPPs to allow more time for the public to engage in an open conversation about variances and to allow people to make an informed decision instead of being coerced—thereby feeling alienated and increasing vaccine hesitancy.

The above analysis helps to accentuate a deeper understanding of the complexities associated with the current polarising public view on VPP and the problematic implementation of VPP—where those in the rebel and entryist groups may oppose while those in the resigned and passive groups may accept VPPs. Understanding the potential types of resistance provides useful strategic responses to mitigate resistance in a constructive manner. For example, based on our earlier analysis of Chaney and Slimane [71], and Hirschman [73], governments should create effective communication channels and provide a conducive atmosphere that allows people to voice their concerns regarding VPPs. Governments should then engage the public by genuinely addressing their concerns with available data and a cogent public health argument instead of trying to suppress public debate and discredit dissenting voices. This is consistent with evidence from Japan, where it is believed that the alienation of anti-vaxxers has increased vaccine hesitancy and created more social problems [36].

## 5. Conclusions

This article adopts an institutional theory perspective (i) to review the barriers responsible for the global vaccine divide and (ii) to explore the arguments of critics and proponents to determine whether vaccine passports are tools for freedom or social exclusion. Our findings suggest that vaccine politics, disruptions in supply chains, fake news, and vaccine fakery and racketeering are inextricably responsible for the global disparities in COVID-19 vaccination. Furthermore, although VPPs provide safer and better tools for the economy than lockdowns, only allowing individuals who are vaccinated to attend large public events or travel internationally can lead to discrimination and social exclusion for certain groups.

Based on our review of consumer resistance and institutional theories, we submit the following policy recommendations. Firstly, governments should explore a persuasive approach to vaccination and VPPs. A useful strategic response is for governments to improve effective communication with the public. This will provide an avenue for an open and informed discussion around the benefits of vaccination as well as the public’s concerns. Voluntary vaccination has been successful in certain countries, including Chile, the UK and the US, where over 60% of the adult population has received at least one shot. Furthermore, government backtracking on public health promises, such as the unauthorised use of digital contact-tracing data for state surveillance (see Section 3), is counterproductive and erodes public trust. For example, a survey of over 13,000 participants across 19 countries reported that, although COVID-19 vaccine acceptance rates vary across countries and regions, countries with high levels of public trust tend to have low levels of vaccine hesitancy [87].

Secondly, while VPPs may not be desirable or acceptable in domestic settings (e.g., local travel, attending local events, etc.) considering potential public resistance and various barriers associated with vaccination, VPPs may be inevitable for international travel. However, such preferences depend upon the public attitude towards COVID-19 or similar risks, concerns towards privacy and cultural context. However, given the existence of multiple vaccines vis à vis vaccine politics and protectionism, some countries may have their vaccine preferences and, by extension, accept only some vaccine passports. The international community should aim toward some form of standardisation to mitigate the lack of mutual VPPs recognition, perhaps through global adoption of the WHO International Certificate of Vaccination [12,88].

Thirdly, given the various reasons for the global vaccine divide, governments should adopt a multi-prong approach to encourage vaccination and not focus solely on VPPs. Hybrid policies that consider a mix of, for example, VPPs, rapid/affordable PCR COVID-19 testing and good hygiene practices would be more viable and attractive to the general public. Therefore, VPPs should be considered as one of the many tools to ‘safely’ reopen the global economy. Similarly, those with underlying medical conditions and disabilities that prohibit COVID-19 vaccination could be given exemption certificates for international travel and access to large public events to mitigate discrimination. The public should also be offered more COVID-19 vaccine options considering the ‘very rare’ side-effects associated with, for example, Oxford-AstraZeneca (blood clot), Pfizer, or Moderna (heart inflammation) [25,89].

Additionally, some critics argue that VPPs are akin to the creation of a ‘permanent solution for a temporary problem’. Thus, governments should be explicitly clear that VPPs are temporary with limited time and scope to allow public health authorities to better understand the long-term effects of COVID-19. Governments should also engage in more public awareness campaigns using digital and print media and ‘trusted’ local community leaders and influencers to provide more information and advance vaccine debates.

Furthermore, governments should ensure that data linked with (digital) vaccine passports are properly stored and not misused by public and private organisations. Moreover, countries could adopt a paper certificate, such as those for yellow fever, to avoid the storage of personal data and the infrastructure requirements associated with digital vaccine passports. Such paper certificates can be produced with security features such as those used for paper money or authenticated by stamps and designated signatures of public health authorities to mitigate forgery.

While these recommendations may not be universally applicable due to dynamic institutional and other resource constraints, they could be adapted to accommodate equity and freewill. VPPs can then be implemented in a way that promotes equality and avoids social exclusion, where one group will be able to enjoy freedom while others linger in COVID-19 restrictions. Moreover, amidst the current dominant Omicron BA.5 strain [90], a growing proportion of the world population is increasingly becoming protected by infection-induced immunity (i.e., natural immunity arising from antibody generated from infection) and/or vaccine-induced immunity (immunity generated from approved vaccination) [91,92,93]. As highlighted in Section 3, while COVID-19 vaccines are effective in breaking the link between serious illness and death, people who are vaccinated can still contract and spread COVID-19 as existing vaccines do not completely stop transmission. Furthermore, an increasing number of countries (apart from China) are now moving away from the ‘zero-COVID-19′ strategy to a more flexible approach to ‘living’ with COVID-19 [53,94,95,96,97,98]. With all these in mind, do governments and policymakers still need to insist on mandatory VPPs?

## Figures and Tables

**Table 1:** Examples of state and non-state actors implementing/planning vaccine passport policies (VPPs).

State Actors		
Country	VPPs Development	Remarks
Canada	Mandatory VPPs effective August 2021	▪ Canada opens its border to fully vaccinated people from the US in August 2021 ▪ Public sector workers across Canada have until end of October 2021 to get vaccinated or risk losing their jobs ▪ Mandatory vaccination required for commercial airline and interprovincial train passengers
EU Member Sates	The EU Digital COVID Certificate effective July 2021	The EU VPPs allow citizens with vaccination, negative test or recovered from COVID-19 to travel across 27 Member States without quarantine and other travel restrictions
France	Mandatory VPPs effective August 2021	▪ Evidence of vaccination, recent recovery from COVID-19 or negative test required to access public places such as cinemas, museums, café, and shopping centres ▪ Businesses who fail to check customers’ status could be shut down for seven days ▪ Vaccination compulsory for all healthcare workers
Israel	VPPs mandated by March 2021	▪ VPPs later scrapped for domestic use by June 2021 following over 90% of adult vaccination
Italy	Mandatory VPPs effective August 2021	▪ Bar public transport, only those (12 years and above) with at least one jab would be able to access venues such as cinemas, gyms and indoor restaurants ▪ Vaccination compulsory for all healthcare workers ▪ VP or negative test required from all workers from October 2021 or face fine up to 1500 euros
Kenya	Mandatory VPPs effective August 2021	▪ Public sector workers across Kenya have until end of August 2021 to get vaccinated or face disciplinary measures from the government
Saudi Arabi	Mandatory VPPs effective August 2021	▪ Umrah pilgrimage to Mecca opened to fully vaccinate ▪ General tourism opened to foreigners who have been fully vaccinated ▪ Currently recognises four vaccines: Johnson & Johnson, Moderna, Oxford-AstraZeneca and Pfizer-BioNTech
UK	Mandator VPPs from the end of September 2021 in England and Scotland for nightclubs and ‘large’ crowd events	▪ VPPs lack harmonization, including the mobile applications across England, Scotland and Wales ▪ All four nations withing the UK issue VPP for international travel ▪ Mandatory vaccination for care home workers and visitors in England ▪ Fully vaccinated travellers from the EU and US are exempted from quarantining in England ▪ Fully vaccinated travellers exempted from PCR tests ▪ Fully vaccinated people and under 18s in England and Northern Ireland exempted from self-isolation ▪ University students across England are being offered cash incentives for vaccination ▪ Some universities across the UK require vaccine passport from graduates and their invitees to attend academic congregation ▪ VPP is required for night clubs and large events in Scotland and Wales
US	VPPs mandatory in selected states, cities and federal agencies from August 2021	▪ Government employees across California and New York City must be vaccinated or get weekly test ▪ Vaccination required to access business and public places in New York ▪ The US Department of Veterans Affairs says its 115,000 frontline health care workers have between August and September 2021 for vaccination or face possible dismissal ▪ The US Defence Department to mandate vaccination for the US military from September 2021 ▪ President Biden announced vaccine mandate for workers in large companies or provide weekly testing
**Non-State Actors**		
English Premier League (EPL)	▪ EPL to randomly ask football fans for prove of full vaccination for 2021/2022 football session ▪ Chelsea FC say supporters must proof full vaccination or negative test to attend live matches	
Facebook	Mandatory vaccination for workers returning to US campuses announced in July 2021	
Google	Mandatory vaccination for workers returning to US campuses with further extension to their global offices. This was announced in July 2021	
JP Morgan	From July 2021, fully vaccinated employees will be exempted from wearing face masks in the US	
Morgan Stanley	Announced in June 2021 that unvaccinated client and employees will not be allowed into its New York office	
Netflix	Mandatory vaccination for cast and crew on US TV and film productions as announced in July 2021	
United Airlines	Mandatory vaccination for employees of the Chicago-based airline or face redundancy	

Sourced from a variety of sources by the authors.

**Table 2:** Highlights from Ipsos MORI’s survey on COVID-19 vaccine passport perceptions.

Issues	Outcome	Implications for Our Paper	Relevance to Institutional Theory
Access to smart devices	51% of people over 65 years lacked access to smartphones	An indication that the digital divide will make the most clinically vulnerable group struggle to access and use a digital COVID-19 VP	The digital divide is a barrier to public acceptance and rejection of VPPs
Discrimination	65% of participants raised discrimination concerns against young people, vulnerable groups such as ethnic minorities, medically shielded individuals and those ineligible for COVID-19 vaccines	This is consistent with one of the arguments against VPPs highlighted in Section 3	Social exclusion can lead to public rebellion
Supporting international travel	73% of participants supported the idea of using VPPs as a tool for resuming international travel and getting industry back on its feet	While VPPs are very divisive for domestic use, people are more receptive of VPPs in the context of international travel as host countries have the right to set their own rules	Despite dissatisfaction, consumers may still tolerate and accept discontent
Reopening the economy and attending large public events	66% of participants supported the use of a VP as a safe tool for reopening the economy; 54% agreed that only the vaccinated should be allowed to attend large public events	While the first part of this finding supports an argument in support of VPPs in Section 3, the second part would lead to discrimination and the ‘two-tiered’ society raised in Section 3	Social exclusion can lead to public rebellion
A catalyst for improving global COVID-19 vaccination	Despite the consensus of VPPs leading to discrimination, over 50% of participants believed that VPPs would increase global COVID-19 vaccination	While an increase in COVID-19 vaccination is useful for meeting population immunity as highlighted in Section 5, it also supports the opposing argument of coercing and indirectly mandating vaccination raised in Section 3	Underlines consumers as passive meaning takers
Banning VPPs	45% of participants oppose the idea of banning VPPs, 22% strongly support their implementation and 33% were undecided	This indicates a lack of clear-cut evidence from the data as to whether VPPs should be banned, further underlining our controversial and divisive premise argument in Section 1	The public may adopt a passive or resigned approach to VPPs; conversely, others may rebel

Note: VP = vaccine passport; VPP = vaccine passport policy. Source: Publicly available data from Ipsos MORI [45].

**Table 3:** Potential reactions to VPPs.

		Opposition to VPPs	
		Yes	No
**Sources of consumption**	**Nonintentional** Consumers want to receive the vaccine but cannot	Entryist	Resigned consumers
	**Intentional** Consumers can receive the vaccine but do not want it	Rebel	Passive consumers

Source: Adapted by the authors from Chaney and Slimane [71] and Slimane et al. [72].

## Data Availability

The data are contained within the article.
